# Prognostic significance of tumor genotypes and CD8+ infiltrates in stage I-III colorectal cancer

**DOI:** 10.18632/oncotarget.26256

**Published:** 2018-11-02

**Authors:** Elena Fountzilas, Vassiliki Kotoula, Ioannis Tikas, Kyriaki Manousou, Kyriaki Papadopoulou, Christos Poulios, Vasilios Karavasilis, Ioannis Efstratiou, Dimitrios Pectasides, Kleo Papaparaskeva, Ioannis Varthalitis, Christos Christodoulou, George Papatsibas, Sofia Chrisafi, Georgios K. Glantzounis, Amanda Psyrri, Gerasimos Aravantinos, Georgia-Angeliki Koliou, George K. Koukoulis, George E. Pentheroudakis, George Fountzilas

**Affiliations:** ^1^ Department of Investigational Cancer Therapeutics, University of Texas MD Anderson Cancer Center, Houston, Texas, USA; ^2^ Department of Pathology, Faculty of Medicine, School of Health Sciences, Aristotle University of Thessaloniki, Thessaloniki, Greece; ^3^ Laboratory of Molecular Oncology, Hellenic Foundation for Cancer Research/Aristotle University of Thessaloniki, Thessaloniki, Greece; ^4^ Section of Biostatistics, Hellenic Cooperative Oncology Group, Athens, Greece; ^5^ Department of Medical Oncology, Papageorgiou Hospital, Faculty of Medicine, School of Health Sciences, Aristotle University of Thessaloniki, Thessaloniki, Greece; ^6^ Department of Pathology, Papageorgiou Hospital, Thessaloniki, Greece; ^7^ Oncology Section, Second Department of Internal Medicine, Hippokration Hospital, Athens, Greece; ^8^ Department of Pathology, Konstantopouleio Agia Olga General Hospital, Athens, Greece; ^9^ Oncology Department, General Hospital of Chania, Crete, Greece; ^10^ Second Department of Medical Oncology, Metropolitan Hospital, Piraeus, Greece; ^11^ Oncology Department, University General Hospital of Larissa, Larissa, Greece; ^12^ Department of Surgery, University Hospital of Ioannina and School of Medicine, University of Ioannina, Greece; ^13^ Division of Oncology, Second Department of Internal Medicine, Attikon University Hospital, Athens, Greece; ^14^ Second Department of Medical Oncology, Agii Anargiri Cancer Hospital, Athens, Greece; ^15^ Department of Pathology, Faculty of Medicine, University of Thessaly, Larissa, Greece; ^16^ Department of Medical Oncology, Ioannina University Hospital, Ioannina, Greece; ^17^ Aristotle University of Thessaloniki, Thessaloniki, Greece

**Keywords:** targeted NGS, CD8, MMR, BRCA1, ARID1A

## Abstract

**Background:**

We explored the clinical significance of tumor genotypes and immunophenotypes in non-metastatic colorectal cancer (CRC).

**Methods:**

In primary tumors (paraffin blocks) from 412 CRC patients treated with adjuvant chemotherapy, we examined pathogenic mutations (panel NGS; 347 informative); mismatch repair (MMR) immunophenotype (360 informative); and CD8+ lymphocyte density (high – low; 412 informative). The primary outcome measure was disease-free survival (DFS).

**Results:**

We evaluated 1713 pathogenic mutations (median: 3 per tumor; range 0-49); 118/412 (28.6%) tumors exhibited high CD8+ density; and, 40/360 (11.1%) were MMR-deficient. Compared to MMR-proficient, MMR-deficient tumors exhibited higher CD8+ density (chi-square, p<0.001) and higher pathogenic mutation numbers (p=0.003). High CD8+ density was an independent favorable prognosticator (HR=0.49, 95%CI 0.29-0.84, Wald's p=0.010). Pathogenic BRCA1 and ARID1A mutations were inversely associated with each other (p<0.001), were not associated with MMR-deficiency or CD8+ density, but both independently predicted for unfavorable DFS (HR=1.98, 95%CI 1.12-3.48, p=0.018 and HR=1.99, 95%CI 1.11-3.54, p=0.020, respectively).

**Conclusion:**

In non-metastatic CRC, high CD8+ lymphocyte density confers a favorable prognosis and may be developed as a single marker in routine diagnostics. The unfavorable prognostic effect of pathogenic BRCA1 and ARID1A mutations is a novel observation that, if further validated, may improve treatment selection.

## INTRODUCTION

During the past decades, the heterogeneity of colorectal cancer (CRC) has become evident [1]. Recently, an international consortium showed that there is significant prognostic variability associated with specific tumor characteristics in colorectal cancer [2]. The revelation of clinically significant, biological characteristics of CRC has become a priority.

Tumor genotypic characteristics have been extensively studied with respect to clinical and pathological tumor heterogeneity and prognosis in patients with metastatic CRC [3–5]. However, their prognostic significance in non-metastatic CRC remains controversial [6–9]. Increasing evidence also suggest that the local adaptive immune response plays a central role in disease recurrence and overall survival (OS) of patients with CRC [10, 11]. There is a need to map the non-metastatic CRC oncogenic mutational and immunophenotypic landscape to accurately stratify patients. The “one-size-fits-all” approach needs to be replaced by personalized management based on the specific molecular alterations of each tumor in early-stage disease.

Our goal was to explore clinicopathological and prognostic significance of tumor molecular alterations and CD8+ lymphocyte density and identify clinically relevant biomarkers in patients with stage I-III CRC. We used targeted next-generation sequencing and immunophenotyping of colorectal tumors to identify clinically relevant genotypic and phenotypic tumor characteristics and assess their associations with patient outcomes.

## RESULTS

### Clinicopathological characteristics

Our study included 412 patients with colorectal cancer, 229 men and 183 women. Median age was 65 years. Based on the histological report, 20% of the tumors were grade 3. The distribution of TNM stage was: 3.7% stage I, 33.4% stage II and 62.9% stage III. Perineural (PNI) and/or lymphovascular (LVI) invasion was noted in 35% of the tumors. R1 resection (microscopic residual tumor) or R2 resection (macroscopic residual tumor) was reported in 3 of 341 patients (0.8%). All patients received adjuvant treatment with chemotherapy, with a median of 8 cycles with or without radiation therapy.

Detailed clinicopathological characteristics are reported in Table 1.

**Table 1:** Clinicopathological characteristics in the entire cohort and by tumor location (right colon, left colon, rectum)

Parameter	Entire cohort (n=412)	Right colon (n=139)	Left colon (n=171)	Rectum (n=102)	p-value
**Age**					0.15
Mean +/− SD	63.1 +/− 10.7	63.5 +/− 10.9	63.8 +/− 10.5	61.5 +/− 10.7	
Median (IQR)	65.3 (56.5, 71.6)	64.9 (56.1, 72.3)	66.3 (57.1, 72.0)	63.0 (53.4, 70.1)	
Min-Max	28-81	28-80	28-81	28-79	
**Gender**					0.953
Female	183 (44.4%)	62 (44.6%)	77 (45.0%)	44 (43.1%)	
Male	229 (55.6%)	77 (55.4%)	94 (55.0%)	58 (56.9%)	
**Tumor location**					N/A
Ascending	62 (15.1%)	62 (44.9%)	0	0	
Cecum	48 (11.7%)	48 (34.8%)	0	0	
Cecum (multifocal)	2 (0.5%)	2 (1.4%)	0	0	
Descending	16 (3.9%)	0	16 (9.4%)	0	
Hepatic flexure	10 (2.4%)	10 (7.2%)	0	0	
Rectosigmoid	8 (1.9%)	0	8 (4.7%)	0	
Rectum	102 (24.9%)	0	0	102 (100.0%)	
Sigmoid	138 (33.7%)	0	138 (81.2%)	0	
Splenic flexure	8 (2%)	0	8 (4.7%)	0	
Transverse	16 (3.9%)	16 (11.6%)	0 (0%)	0	
Missing	2				
**Perforation**					**0.017**
No	375 (96.4%)	129 (98.5%)	151 (93.2%)	95 (99.0%)	
Yes	14 (3.6%)	2 (1.5%)	11 (6.8%)	1 (1.0%)	
Missing	23				
**Obstruction**					**0.015**
No	345 (88.5%)	118 (90.1%)	135 (83.3%)	92 (94.8%)	
Yes	45 (11.5%)	13 (9.9%)	27 (16.7%)	5 (5.2%)	
Missing	22				
**Primary tumor (T)**					N/A
T1	4 (1.0%)	0	2 (1.2%)	2 (2.0%)	
T2	35 (8.5%)	8 (5.8%)	12 (7.1%)	15 (15.0%)	
T3	329 (79.9%)	117 (84.2%)	136 (80.0%)	76 (76.0%)	
T4	41 (10.0%)	14 (10.1%)	20 (11.8%)	7 (7.0%)	
Missing	3				
**Regional lymph nodes (N)**					0.542
N0	153 (37.5%)	56 (40.3%)	60 (35.7%)	37 (36.6%)	
N1	176 (43.1%)	56 (40.3%)	71 (42.3%)	49 (48.5%)	
N2	79 (19.4%)	27 (19.4%)	37 (22.0%)	15 (14.9%)	
Missing	4				
**Stage, detailed categories**					N/A
I	15 (3.7%)	6 (4.3%)	6 (3.5%)	3 (3.0%)	
IIA	122 (29.7%)	43 (30.9%)	46 (27.1%)	33 (32.7%)	
IIB	15 (3.7%)	6 (4.3%)	8 (4.7%)	1 (1.0%)	
IIIA	27 (6.6%)	6 (4.3%)	10 (5.9%)	11 (10.9%)	
IIIB	153 (37.3%)	52 (37.4%)	63 (37.1%)	38 (37.6%)	
IIIC	78 (19.0%)	26 (18.7%)	37 (21.8%)	15 (14.9%)	
Missing	2				
**Grade**					**0.001**
1	21 (5.3%)	7 (5.2%)	6 (3.6%)	8 (8.1%)	
2	299 (74.9%)	86 (64.2%)	131 (78.9%)	82 (82.8%)	
3	79 (19.8%)	41 (30.6%)	29 (17.5%)	9 (9.1%)	
Missing	13				
**Mucinous component**					0.21
No	273 (72.8%)	84 (67.2%)	120 (76.4%)	69 (74.2%)	
Yes	102 (27.2%)	41 (32.8%)	37 (23.6%)	24 (25.8%)	
Missing	37				
**Perineural invasion**					0.576
No	328 (84.6%)	110 (87.3%)	135 (82.8%)	83 (84.7%)	
Yes	59 (15.4%)	16 (12.7%)	28 (17.2%)	15 (15.3%)	
Missing	25				
**Lympatic vessel invasion**					0.43
No	298 (77.0%)	92 (73.0%)	129 (79.1%)	77 (78.6%)	
Yes	89 (23.0%)	34 (27.0%)	34 (20.9%)	21 (21.4%)	
Missing	25				
**Blood vessel invasion**					0.543
No	332 (86.0%)	109 (86.5%)	136 (84.0%)	87 (88.8%)	
Yes	54 (14.0%)	17 (13.5%)	26 (16.0%)	11 (11.2%)	
Missing	26				
**Treatment**					0.238^*^
Capecitabine	1 (0.2%)	0	0	1 (1.0%)	
FOLFOX	137 (33.6%)	40 (29.0%)	64 (38.1%)	33 (32.4%)	
CAPOX	270 (66.2%)	98 (71.0%)	104 (61.9%)	68 (66.7%)	
Missing	4				
**Radiation therapy**					N/A
No	279 (76.9%)	119 (99.2%)	147 (97.4%)	13 (14.1%)	
Yes	84 (23.1%)	1 (0.8%)	4 (2.6%)	79 (85.9%)	
Missing	49				

^*^1 patient with Capecitabine was excluded from the estimation of p-value.

Abbreviations: IQR: interquartile range, LVI: lymphovascular invasion, n: number, No: number, N/A: not applicable, PNI: perineural invasion, PS: performance status, SD: standard deviation.

Tumors were located at the right colon (139, 34%), left colon (171, 41%) or rectum (102, 25%). Stage distribution (I, II, III) did not differ between the different tumor locations (chi-square p=0.937). A significant association was observed between tumor location and grade (chi-square p=0.001), with right-sided tumors being more frequently high-grade (30.6%) compared to left-sided (17.5%) or rectal tumors (9.1%).

### Mismatch repair (MMR) immunohistochemistry (IHC) status and immune cell infiltrates

The distribution of IHC parameters in the entire cohort and by tumor location are presented in Table 2. Among the 360 tumors with informative MMR IHC status, 40 exhibited MMR deficiency (MMR-D, 11.1%). MMR-D was significantly more frequent in right-sided tumors (25.8%) compared to left-sided (4%) and rectal tumors (3.3%) (chi-square, p<0.001). In comparison to MMR-proficiency (MMR-P), MMR-D was associated with younger age (Mann-Whitney p=0.010); higher histological grade; grade 3 in 42.1% MMR-D and 17.2% in MMR-P tumors (chi square p<0.001), and lower disease stage; stage III was diagnosed in 47.2% patients with MMR-D compared to 64.6% patients with MMR-P tumors (chi-square, p=0.041). The distribution of clinicopathological characteristics according to tumor MMR status is shown in Supplementary Table 1.

**Table 2:** IHC and NGS parameters in the entire cohort and by tumor location (right colon, left colon, rectum)

Parameters^*^	Entire cohort (n=412)	Right colon (n=139)	Left colon (n=171)	Rectum (n=102)	p-value
			**IHC**		
**MMR status**					**<0.001**
Deficiency	40 (11.1%)	31 (25.8%)	6 (4.0%)	3 (3.3%)	
Proficiency	320 (88.9%)	89 (74.2%)	144 (96.0%)	87 (96.7%)	
Missing	52				
**CD8+ density**					**<0.001**
High^	118 (28.6%)	57 (41.0%)	32 (18.7%)	29 (28.4%)	
Other^	294 (71.4%)	82 (59.0%)	139 (81.3%)	73 (71.6%)	
	**NGS**					
**BRAF**					**0.020**
No	321 (92.5%)	99 (86.8%)	141 (95.3%)	81 (95.3%)	
Yes	26 (7.5%)	15 (13.2%)	7 (4.7%)	4 (4.7%)	
**TP53**					**0.013**
No	158 (45.5%)	61 (53.5%)	54 (36.5%)	43 (50.6%)	
Yes	189 (54.5%)	53 (46.5%)	94 (63.5%)	42 (49.4%)	
**FBXW7**					**0.002**
No	309 (89.0%)	104 (91.2%)	138 (93.2%)	67 (78.8%)	
Yes	38 (11.0%)	10 (8.8%)	10 (6.8%)	18 (21.2%)	
**PALB2**					**0.026**
No	321 (92.5%)	107 (93.9%)	141 (95.3%)	73 (85.9%)	
Yes	26 (7.5%)	7 (6.1%)	7 (4.7%)	12 (14.1%)	
**PIK3CA**					**0.018**
No	284 (81.8%)	84 (73.7%)	129 (87.2%)	71 (83.5%)	
Yes	63 (18.2%)	30 (26.3%)	19 (12.8%)	14 (16.5%)	

^*^analysis was performed in informative samples.

Abbreviations: IHC: immunohistochemistry, IQR: interquartile range, MMR: mismatch repair, n: number, MUT: mutation, NGS: next-generation sequencing, N/A: not applicable, SD: standard deviation; ^: High: high CD8+ density, stromal AND intratumoral (in direct contact with cancer cells); Other: only stromal high OR only intratumoral high OR none high.

We also assessed the density of CD8+ cells in the tumor center and front, distinguishing for CD8+ in the tumor stroma (stromal) and within cancer cell nests, in direct contact to cancer cells (intratumoral). However, as described in detail in Methods, we finally evaluated the combined presence of CD8+ independently of tumor compartment as “high”, if stromal and intratumoral CD8+ density were above the corresponding cut-offs for each architectural compartment; and “other” for all other combinations. A significant association was observed between CD8+ density and tumor location. Right-sided tumors more frequently had high CD8+ density compared to the other tumor sites (CD8+ high: right-sided 41%, left-sided 19% and rectal tumors 28%, chi-square, p<0.001) (Table 2).

In comparison to MMR-P tumors, MMR-D tumors were more likely to exhibit high CD8+ density (65% in MMR-D vs. 27% in MMR-P, chi-square, p<0.001). Details are shown in Supplementary Table 1.

### NGS results

We identified a total of 5239 mutations in 55 genes distributed in 339 out of 347 NGS informative tumors (median 4; range 1 – 220; mean ± SD 15±36). Out of all mutations, in the same genes, 1713 were pathogenic and were found in 332/347 (95.7%) tumors. The median number of pathogenic mutations per tumor was 3 (range, 1-49 mutations). The median number of genes with pathogenic mutations per tumor was 3 (range, 1-28 genes). Tumors without mutations (N=8) and without pathogenic mutations (N=15) in the panel genes were considered as true negatives, based on their sequencing characteristics (Supplementary Figure 1). The median number of mutations, pathogenic mutations and mutated genes per tumor did not differ between tumor locations (Mann-Whitney, p=0.460, p=0.520, p=0.620, respectively).

The distribution and characteristics of mutations are shown in Figure 2A, and the prevalence of pathogenic mutations in Figure 2B. Among the 347 tumors, pathogenic mutations most frequently concerned APC (56%), TP53 (55%), KRAS (48%), PIK3CA (18%), and BRCA1 (13%). BRAF mutations were present in 26/347 tumors (7.5%) (Figure 2B); 21 had the classic p.Val600Glu (p.V600E); 4 had non-V600E mutations; and, 1 tumor had p.Val600Glu and p.Ala598Thr at respective variant allelic frequencies (VAFs) of 16% and 10% in a sample with 50% tumor cell DNA (Supplementary Table 2). Mutations in the 4 MMR genes encoding the proteins assessed by IHC (MLH1, PMS2, MSH2, MSH6) were identified in 22 tumors (6.3%). MMR gene mutations and IHC MMR status was comparable in 17 tumors. MMR-D was observed in only 4 of these tumors, including one case with double MLH1 and MSH2 protein loss and corresponding mutations; the remaining 13 tumors with MMR gene mutations were characterized as MMR-P with IHC (Supplementary Table 3).

**Figure 1:**
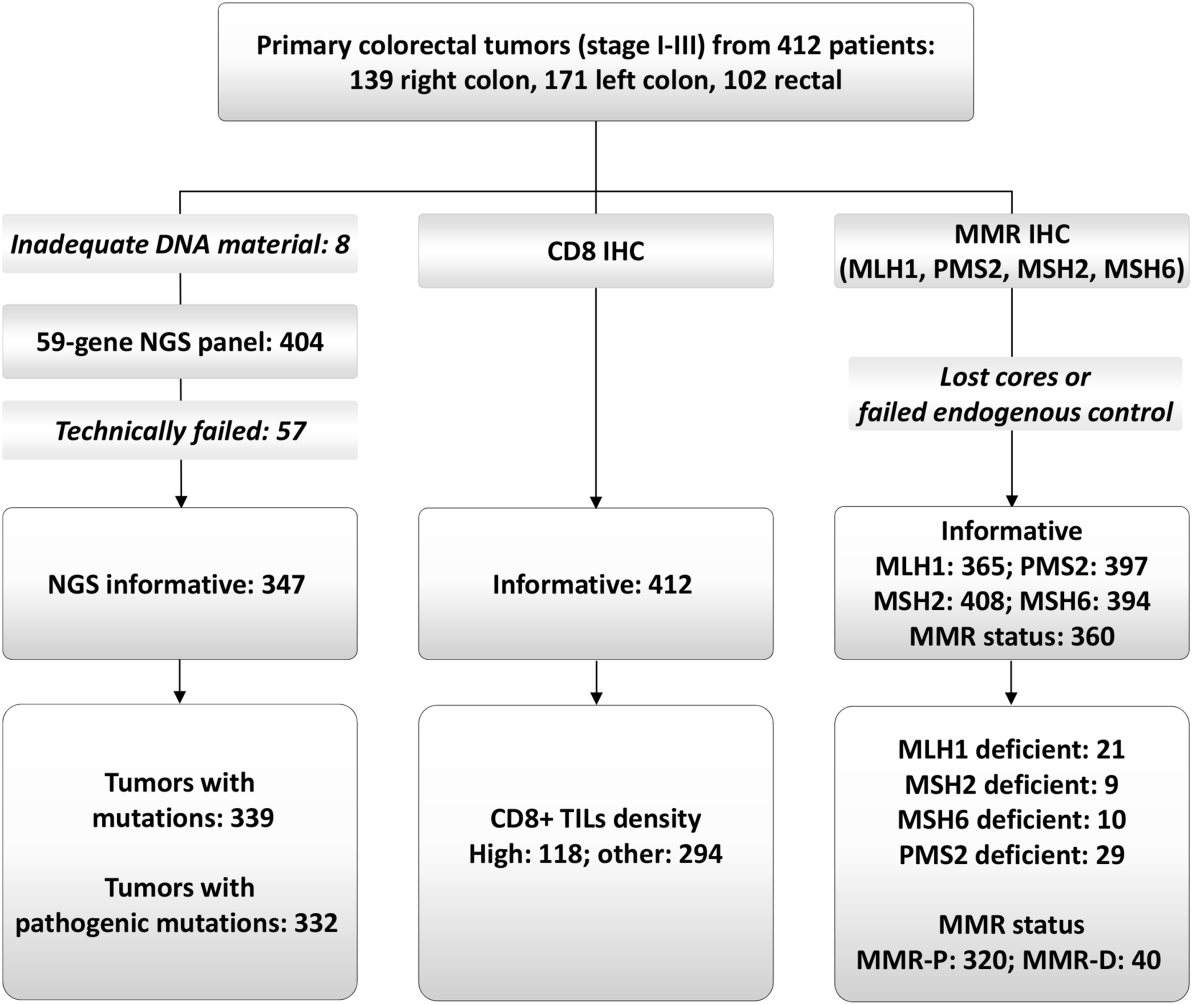
REMARK diagram NGS: next generation sequencing; MMR: mismatch repair; MMR-P and MMR-D: proficient and deficient, respectively.

**Figure 2:**
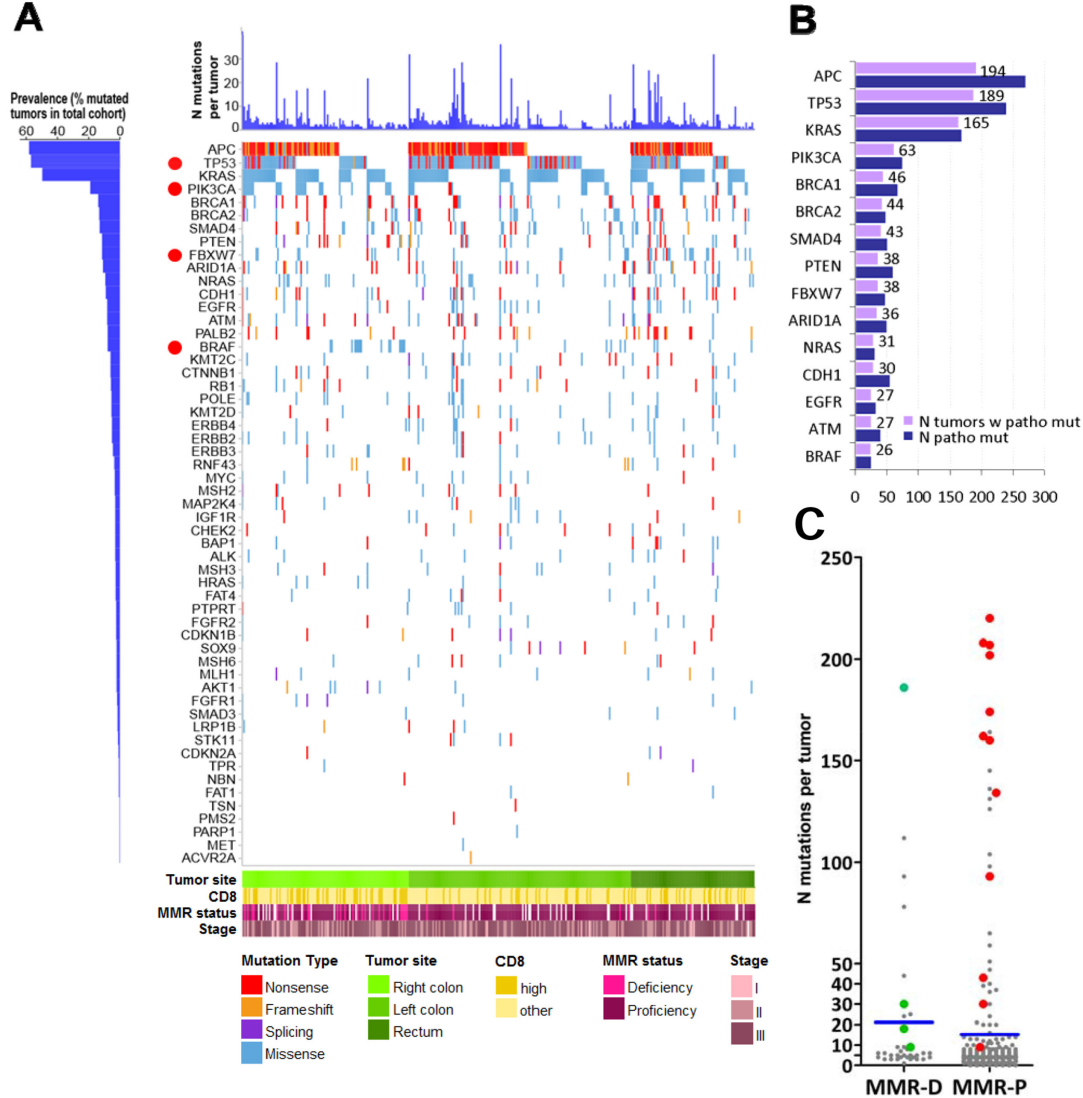
Map of pathogenic mutations in 332 CRC **(A)** Out of 1713 pathogenic mutations, 32% were nonsense or frameshifts in tumor suppressors, while missense mutations were dominant in known oncogenes. We did not apply the classification of hypermutated and non-hypermutated tumors because we used a 59-gene panel only. However, it is apparent that most tumors (75%) carried more than 1 pathogenic mutation, the most frequent combination being APC & TP53 in 1/3 of tumors, co-mutated with KRAS in ¼ of the cases, while 10% of tumors carried more than 10 pathogenic mutations. Despite that the applied reading depth was very high in our cases (>1000X, compared to <50X in whole genome sequencing), the 4 most frequently mutated genes are in line with previous publications. The high incidence of BRCA1, PTEN, CDH1 and BRCA2 mutations is most probably a result of high reading depth and over-representation of these genes in the custom panel. Red dots: genes with site-specific differences in the distribution of pathogenic mutations. **(B)** Demonstrates the actual number of tumors with pathogenic mutations in the presented genes. The number of tumors with pathogenic mutations is shown for the 15 most frequently affected genes. Blue bars correspond to the number of pathogenic mutations per gene; tumor suppressor genes, e.g., APC, TP53, occasionally carried multiple mutations per tumor, which was not observed for oncogenes, e.g., KRAS, BRAF. **(C)** Comparison of mutation numbers in MMR-D and MMR-P tumors. Although MMR-D were in general richer in mutations compared to MMR-P tumors, MMR-P tumors with mutations in MMR genes (red dots) exhibited higher mutation numbers compared to MMR-D, probably because of co-mutated pathways. Green dots: four MMR-D tumors with concordant MMR gene mutation status. Blue lines: mean values.

### Associations between mutations and clinicopathological parameters

A significantly higher number of total mutations was noted in MMR-D compared to MMR-P tumors (Mann-Whitney p=0.003) (Figure 2C, Supplementary Table 1). However, compared to tumors identified as MMR-D with IHC, MMR-P tumors with MMR gene mutations in fact exhibited significantly higher numbers of mutations (Mann-Whitney p<0.0001; Figure 2C), were preferentially left sided (11/13 tumors; chi-square p<0.0001); exhibited different patterns of coexisting clonal pathogenic mutations, e.g., a higher incidence in POLE (Fisher's exact p=0.0002), and a lower incidence in the RAS/RAF pathways (Fisher's exact p=0.0155) (Supplementary Table 3).

There was no association between mutations in the most frequently affected genes and CD8+ density. Most of the tumors with ATM (p=0.043) and BRAF (p=0.015) mutations had high CD8+ but with one-sided significance.

BRAF and PIK3CA mutations were more frequently noted in right-sided compared to left-sided colon and rectal tumors (chi-square, p=0.020 and p=0.018, respectively). We observed a higher frequency of FBXW7 mutations in rectal tumors compared to the rest of the tumors (chi square, p=0.002). Left-sided tumors had a higher TP53 mutation rate, compared to right-sided and rectal tumors (chi square, p=0.013) (details in Table 2). RAS mutation rates did not differ among the tumors of the three anatomic locations (chi square, p=694).

Pathogenic BRCA1 and ARID1A mutations were inversely associated with each other (p<0.001). There were 46 patients with BRCA1 and 36 with ARID1A mutated tumors, equally distributed in right, left colon and rectum. Clonal mutations were present in 11 (24%) of BRCA1 and in 16 (44%) of ARID1A mutated tumors, while loss-of-heterozygosity inferred from the frequency of the mutated allele (see Supplementary Methods) was observed in 4 and 7 non-overlapping tumors, respectively. Mutations in BRCA1 and ARID1A were present in tumors with higher numbers of mutations and mutated genes (Mann-Whitney, p's <0.001). Mutations in these genes were mostly present in tumors without mutations in MMR genes (chi-square, p's <0.001), while ARID1A mutations were more frequent in tumors with a mucinous component (chi-square, p=0.005) (Supplementary Table 4). There was no association betweenBRCA1 or ARID1A mutations with MMR-D or CD8+ high density.

### Patient outcomes

During a median follow-up of 87.9 months (range 0.7-125.9), 113 disease-free survival (DFS) events occurred; median DFS was not reached. The effect of clinicopathological characteristics on DFS is presented in Supplementary Table 5. No significant difference in DFS was observed between patients treated with the two chemotherapy regimens (log-rank, p=0.630). Tumor location was not associated with DFS. Upon adjusting for clinicopathological parameters, stage, grade and LVI remained of independent prognostic significance (Supplementary Table 6).

**Figure 3:**
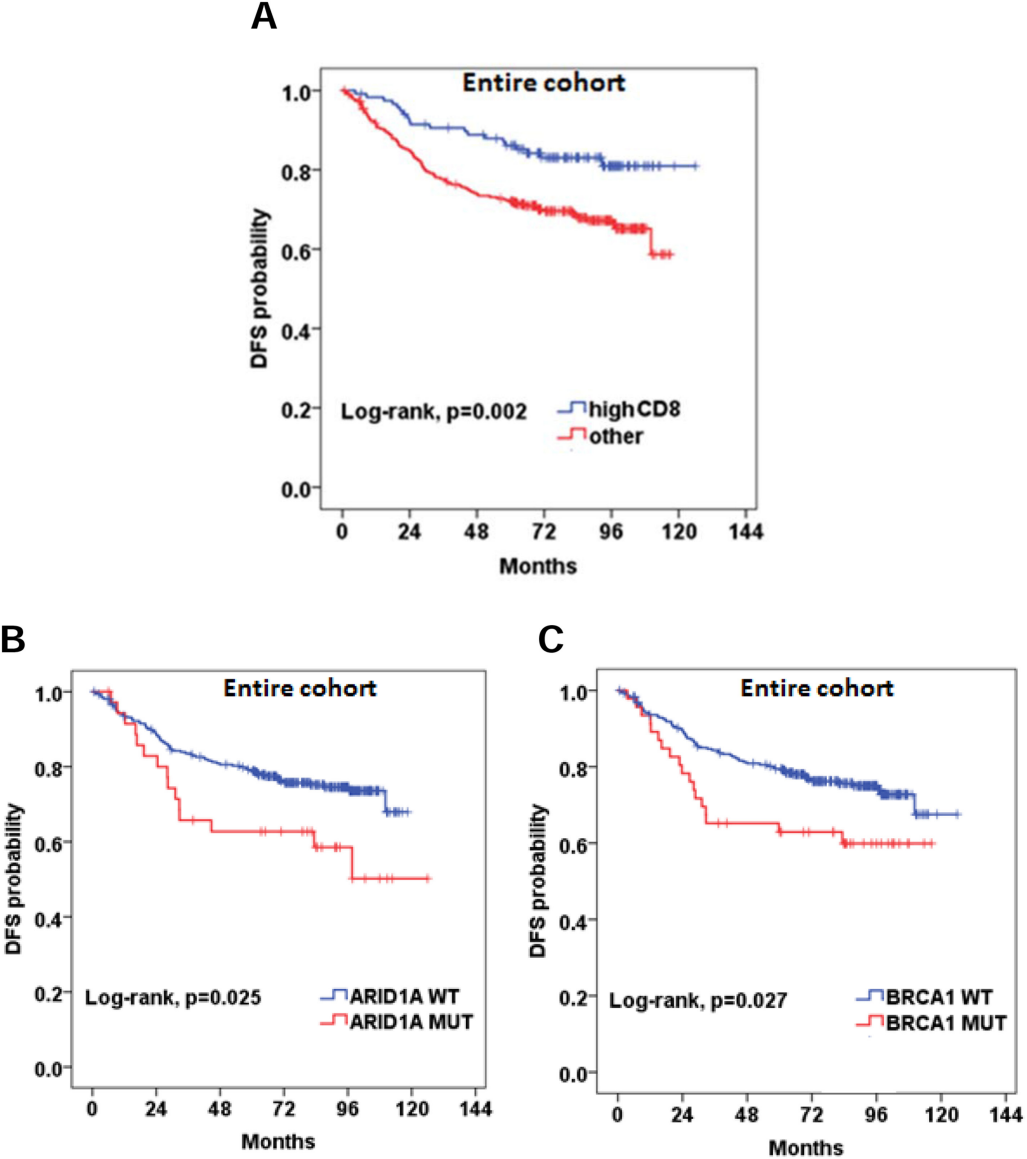
Prognostic significance of **(A)** high CD8+ density; **(B and C)** BRCA1 and ARID1A pathogenic mutations.

Detailed results of the univariate analysis of all study variables are shown in Supplementary Table 7. MMR-D did not appear to be prognostic in the entire cohort (HR=0.61, 95% CI 0.29-1.25, Wald's p=0.170). However, MMR-D was found to be associated with improved DFS in the subgroup of patients with tumors located in the right colon, although the association was of marginal statistical significance (HR=0.36, 95% CI 0.13-1.04, Wald's p=0.058). High CD8+ density (HR=0.48, 95% CI 0.29-0.77, Wald's p=0.003) appeared to be favorably associated with DFS in the entire cohort (Figure 3A).

**Figure 4:**
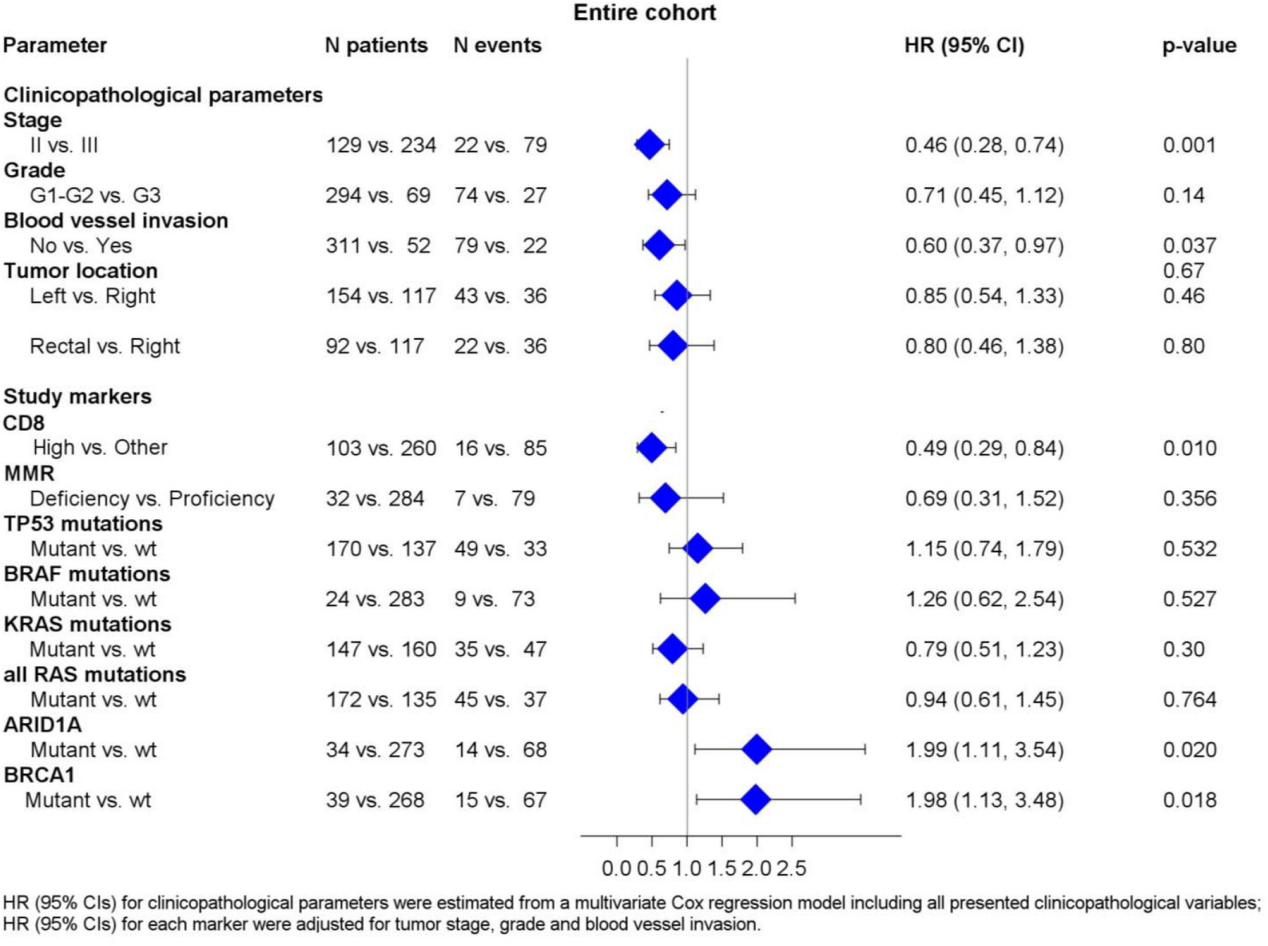
Associations between DFS and relevant clinicopathological, mutational and immunophenotypic parameters

We did not identify any significant association between RAS mutational status and DFS. BRAF mutational status did not appear to be prognostic either in the entire cohort (HR=1.49, 95% CI 0.77-2.88, p=0.239) or in the subgroups of patients with MMR-D and MMR-P tumors (HR=2.63, 95% CI 0.53-13.2, p=0.240 and HR=2.13, 95% CI 0.93-4.93, p=0.076, respectively). Patients with BRAF non-V600E mutations had an excellent outcome, while the only patient with double BRAF p.V600E and non-V600E experienced a relapse at 83 months. BRCA1 and ARID1A, were found to be prognostic in the entire cohort (HR=1.77, 95% CI 1.06-2.97, p=0.030 and HR=1.87, 95% CI 1.07-3.25, p=0.028, respectively) (Figure 3B and 3C). Multivariate analyses were performed in the entire cohort adjusting for tumor stage, grade and blood vessel invasion (Supplementary Table 7). High CD8+ density retained its favorable prognostic significance for DFS (HR=0.49, 95% CI 0.29-0.84, Wald's p=0.010). BRCA1 and ARID1A mutations also retained their prognostic significance in multivariate analyses. The HR's for all relevant study parameters are depicted as a forest plot, in Figure 4.

## DISCUSSION

We demonstrated that non-metastatic colorectal tumors have distinct clinicopathological, mutational and immunophenotypic profiles. We identified prognostic markers to aid in the stratification of non-metastatic CRC patients. High density of tumor infiltrating CD8+ lymphocytes, was associated with good prognosis. Pathogenic mutations in BRCA1 and ARID1A were associated with poor outcomes in the patients of our study.

Recently, a lot of interest has focused on the immunogenic profile of colorectal tumors. The prognostic significance of TILs has been clearly shown in many different studies [12–16]. Moreover, published data suggest that the prognostic significance depends on the specific immune cell types that infiltrate the tumor and the tumor area [11]. Here, we evaluated the prognostic effect of cytotoxic T cells assessed by CD8 within the tumor nests and in the stroma. In accordance to our results, CD8+ density has been previously shown to be a major favorable prognostic factor in CRC [17], and has been successfully used in prognostic immunological profiles [10]. However, there are limited data on the association of CD8+ cell density and tumor location. In our study, right-sided tumors were enriched for high CD8+ density, which may be attributed to the higher rates of MMR-D in those tumors and, therefore, increased immunogenicity. Due to the small sample size of MMR-D tumors, these results need to be addressed with caution. We have previously published an immune response gene expression profile and its stage- and site-specific prognostic implications [18]. A low immune response was associated with inferior DFS only in patients with stage III right colorectal tumors. The novelty of our finding is that the CD8+ cytotoxic T cell density can be used as a single marker of good prognosis, independently of tumor compartment, i.e., core or invasive margin, in non-metastatic stage CRC. Since the morphological assessment of TILs is not reliable in CRC [17], using one IHC marker instead of two or more, in sections where the invasive margin is not assessable, will facilitate the integration of immunodiagnostics in stages I-III of this disease.

It is of high importance to map the genomic profile of CRC, to identify potential prognostic markers or even therapeutic targets. In our patient cohort, we noted differences in mutation rates of specific genes depending on tumor location. Left-sided tumors were more likely to harbor TP53 mutations, also noted in another study [19]. Rectal tumors were enriched for FBXW7 mutations compared to the rest of the tumors, which has also been noticed previously [20, 21]. FBXW7 is a tumor suppressor gene [22], shown to target several proteins implicated in cell division and cell growth, for ubiquitination and subsequent degradation [23–25]. However, the prognostic relevance of rectal FBXW7 mutations remains unclear. Right-sided tumors had a higher frequency of BRAF and PIK3CA mutations compared to left-sided and rectal tumors. Similarly, other investigators showed that right-sided tumors had a higher frequency of PIK3CA mutations [26]. The higher frequency of BRAF mutations has been associated with stage IV right-sided tumors [27]. Emerging data show that BRAF mutations are similarly more frequent in non-metastatic right-sided tumors [28–30]. We have also shown that the mere presence of tumor MMR gene mutations is not necessarily accompanied by MMR protein deficiency. This was not unexpected, since both alleles need to become inactivated for protein loss [31]. Unfortunately, no germline data were available in our patients; thus, we cannot provide any information on the inherited status of the observed MLH1, MSH2 and MSH6 mutations, even in the 4 cases with concordant loss of the corresponding protein. Interestingly, compared to MMR-D, these tumors with MMR gene mutations and MMR-P status, were more likely hypermutated, had different clonal pathogenic mutational profiles in POLE (another gene associated with hypermutation [32] and in the RAS/RAF pathway, and, they were primarily located in the left colon. These features are in line with the recently published profiles of gastrointestinal adenocarcinomas [33]. In addition, mutations in genes traditionally regarded as sources of hypermutation may in fact be surrogates for multiple mutational processes operating in a tumor [34]. Clearly, the presented MMR-P & MMR-mutated phenotype is an exploratory finding in a small number of cases, but it appears worth validating in larger series for comprehending the biology and planning appropriate treatments for these tumors.

BRAF mutations have been extensively studied in CRC, and have been strongly associated with poor outcomes in metastatic tumors [27], particularly with respect to the classical BRAF p.V600E; non-V600E mutations seem to confer favorable prognosis [35], which was indicated in the few such patients in our series as well. In non-metastatictumors, data supporting the prognostic significance of BRAF mutations are not as clear [9, 30, 36–38]. A retrospective analysis of BRAF mutations in prospectively collected tumor blocks from patients enrolled in the PETACC-8 trial demonstrated that the BRAFV600E mutation was not prognostic in the entire cohort [39]. However, subgroup analysis showed that in patients with microsatellite-stable tumors BRAF mutation was independently associated with poor clinical outcomes. Even though BRAF mutational status was not associated with DFS in our cohort, we observed a statistical trend for the association of BRAF mutations with poor prognosis in the subgroup of patients with MMR-P tumors. However, due to the small number of patients with MMR-P/BRAF-mutated tumors (13 patients), we cannot draw definitive conclusions. Another study suggested that the prognostic significance of BRAF mutations depends on the microsatellite instability of the tumor [38]. In contrast, other investigators have shown that the OS between patients with stage I-II CRC with and without BRAF mutations was similar [30]. Further prospective studies are needed to provide robust data on the prognostic significance of BRAF mutations.

We demonstrated that BRCA1 and ARID1A pathogenic mutations were associated with poor DFS in patients with non-metastatic CRC. ARID1A, a tumor suppressor gene, has been suggested as the most commonly deregulated ATP-dependent chromatin remodeler [40]. Studies suggest that loss of ARID1A expression is associated with poor differentiation, higher stage, distant metastasis [41] and lymphovascular invasion [42]. However, the prognostic significance of ARID1A in colorectal cancer has yet to be determined. Other studies supported the prognostic significance of BRCA1 in colorectal cancer. In a retrospective study, loss of heterozygosity (LOH) in the BRCA1 locus was associated with decreased DFS and OS [43]. In another study, high expression of BRCA1 cytoplasmic expression was associated with favorable OS in digestive system cancers [44]. Our findings are in line with these studies. Of note, mutations in these genes were mostly represented at subclonal frequencies within the affected tumors, while LOH, which would imply loss of gene function, was inferred in only 9% of BRCA1 and in 19% of ARID1A mutated tumors. These features and the respective higher mutational burden may suggest that BRCA1 and ARID1A mutations be surrogates for underlying mutational processes that affect CRC behavior [32]. Nevertheless, due to the exploratory nature of our study, these results are hypothesis generating and need to be compared with corresponding deep sequencing results from large patient series and prospectively tested for their clinical value.

Tumor location was not an independent prognostic factor in our patient cohort. Data regarding the independent prognostic significance of tumor location in non-metastatic CRC are conflicting [45–48]. In a retrospective study of 6,365 patients with stage I to III colon cancer, there was no difference in overall and cancer-specific survival between patients with right and left-sided tumors [47]. Population analysis of 91,416 patients with colon cancer demonstrated that compared to left-sided tumors, right-sided colon tumors had significantly increased cancer-specific survival in localized disease (stage I and II) [48]. Cancer-specific survival was equivalent between patients with right- and left-sided tumors in regional disease (stage III). These contradictory data underline the importance of identifying prognostic biomarkers which drive the disparate disease outcomes of patients with stage I – III disease, irrespectively of tumor location.

Our work has certain limitations. First, its retrospective design. Second, our study included patients with stages I to III. Even though we adjusted for stage, there might be molecular differences associated with more advanced disease, which might have confounded our analysis. Third, our NGS panel targeted 59 genes only; therefore, we could not accurately distinguish tumors into hypermutated and non-hypermutated. Finally, the sample size of the study did not allow for assessing the possible prognostic role of genes less frequently mutated.

In conclusion, colorectal tumors have complex clinicopathological, mutational and immunophenotypic profiles. CD8+ density, BRCA1 and ARID1A mutations were shown to be independently associated with DFS in our patient cohort.

CD8+ IHC may be developed as a single marker for integration in routine diagnostics. The clinical impact of these biomarkers, if further validated, may aid in the accurate prognostic stratification of non-metastatic CRC patients. Further studies are needed to comprehend the underlying biological heterogeneity of colorectal tumors and personalize patient management.

## MATERIALS AND METHODS

### Patients and tissues

We retrospectively assessed patients with primary colorectal adenocarcinomas, diagnosed between March 2007 and September 2012, treated in Academic Institutions and private clinics affiliated with the Hellenic Cooperative Oncology Group (HeCOG). Patients were diagnosed with non-metastatic disease (stages I – III) and were followed for at least five years. All patients underwent surgical resection of their primary tumor and then received adjuvant treatment, if needed, depending on clinical and histopathological risk factors. Adjuvant chemotherapy comprised of oxaliplatin, leucovorin and 5-fluoruracil administered intravenously (FOLFOX) or oral capecitabine combined with oxaliplatin administered intravenously (CAPOX). Patients with rectal cancer received adjuvant treatment with chemotherapy and/or radiation therapy, based on the treating physician's judgment. We retrieved patient clinical demographics, tumor histopathological and treatment data from the patients' medical records. Signed informed consent was obtained from all patients for the use of their biologic material for research purposes. The translational protocol was conducted in agreement with the Declaration of Helsinki and was approved by the Institutional Review Boards of “Papageorgiou” Hospital (1338/12-1-2015) and “Thermi” Clinic (307/2-3-2016).

Formalin-Fixed Paraffin-Embedded (FFPE) tissues were retrieved from the HeCOG repository. Central tumor histology review, tissue processing, immunophenotyping and targeted next generation sequencing (NGS) genotyping were performed in the Laboratory of Molecular Oncology (Hellenic Foundation for Cancer Research/Aristotle University of Thessaloniki). We constructed 34 low-density tissue microarrays (TMAs) with multiple 1mm cores per tumor (range per tumor: 3 – 10 cores). Cores from the tumor center were available in 281 and from the tumor front (invasive margin) in 285 tumors (≥4 cores in these cases). No distinction between center and front was possible in 132 cases (3 cores per tumor in these cases). TMAs were used for the application of IHC and NGS. As described in Figure 1, we examined 412 tumors from an equal number of patients.

### IHC methods and interpretation

We performed IHC for CD8+ and MMR proteins (MLH1, PMS2, MSH2, MSH6) on 3μm TMA sections (method details in Supplementary Methods).

We evaluated intratumoral (i-CD8+) and stromal CD8+ (s-CD8+) tumor-infiltrating lymphocyte (TIL) density. i-CD8+ cells were those in direct contact to cancer cells within neoplastic nests. We assessed s-CD8+ cells as area percentage of the entire stromal area by counting CD8+ cells in all medium power fields (magnification X100) on all available TMA cores per tumor, and i-CD8+ cells as percentage of all cells within cancer nests in each of high power field (HPF, magnification X400); i-CD8+ counts were obtained from at least 8 HPFs for tumor core and similarly for tumor front, and from more than 10 HPFs in the cases where compartment distinction was not available. For each tumor, we processed the maximal counts per variable [49], initially for tumor center (278 informative tumors with 2 cores per compartment) and tumor front (276 informative tumors). The distribution of the obtained values between tumor center and front did not vary, as shown in Supplementary Figure 2. Based on this observation, we merged center and front values, again by using the maximal value per paired counts. Based on the distribution of the merged values (Supplementary Figure 2), we categorized (a) s-CD8+ as high (≥15%) and low (<15%), and (b) i-CD8+ as high (≥2%) and low (0-1%).

We then combined these two variables into i-&s-CD8 with initially 4 Cartesian categories (both high, both low, i-high/s-low, i-low/s-high). Because there were only 29 tumors with i-high/s-low (7% of the cohort) which would compromise statistical analysis, and also because we did not observe any difference in the outcome of patients with i-low/s-high (N=106 [25.7%]) and both low (N=159 [38.6%]), we next merged these 4 i-&s-CD8+ categories into both high (N=118 [28.6%]) and all other. The “both high” category corresponded to high density CD8+ cytotoxic T cells in the stroma and in direct contact with cancer cells. The “other” category included high CD8+ density only in the stroma or only among cancer cells or low values in these two compartments.

For the four MMR proteins, intensity and percentage were recorded; markers were evaluated in comparison to internal controls (stromal and endothelial cells, lymphocytes) as: positive, if ≥10 % positive nuclei with mild to strong intensity were counted; negative, if internal controls were positive and tumor cells were completely negative or exhibited any staining <10%; non-informative, if tumor cells were negative and internal controls were negative (assay failure; biallelic loss of the particular protein could not be considered). Tumor MMR status was evaluated if informative results for all four MMR proteins were available. Tumors with negative result in one of the four proteins were classified as MMR deficient [50].

### NGS genotyping and mutation characterization

Method details are provided in Supplementary Methods. Briefly, DNA was extracted from TMA core sections, quality assessed, and submitted for semiconductor sequencing with a custom Ampliseq panel (Thermo – Fisher Scientific, Paisley, UK). Panel targets are shown in Supplementary Table 8. Upon very stringent filtering for minimizing false positive variants, we obtained informative results for 347 out of 404 sequenced tumors. Informative tumors were read at very high depth (median and mean values of mean depth were 1284 and 1659, respectively; technical characteristics and variant distribution are shown in Supplementary Figure 3). Amino acid or splice site changing variants with minor allele frequency <0.1% were called mutations. Based on the obtained mutation frequencies (VAFs), functions and genotypes (Supplementary Figure 4), we analysed only pathogenic or likely pathogenic mutations according to FATHMM, ClinVar and COSMIC. We also assessed mutation clonality based on VAFs compared to tumor cell content (details in Supplementary Methods).

### Statistical analysis

Possible associations between two categorical variables were assessed with the chi-square test. The Mann-Whitney or Kruskal-Wallis tests were used for comparing the values of a continuous variable across the levels of a categorical variable. The primary endpoint was DFS, defined as the time from the date of diagnosis to documented first relapse, death or last contact, whichever occurred first. Surviving patients were censored at the date of last contact. Survival curves were estimated using the Kaplan-Meier method and compared across groups with the log-rank test. The associations between the factors examined and relapse rates were evaluated with hazard ratios, estimated with Cox proportional hazards model. In multivariate analyses, we estimated the effect (HR) of IHC and NGS parameters adjusted for the effect of clinical factors which were univariately associated with DFS. Cox regression analyses including an interaction term between tumor location and selected IHC/NGS parameters were also performed in order to identify factors that differentiated the effect of tumor location on DFS. Because this study was exploratory with predefined parameters, we did not apply correction for multiple testing, based on Feise et al [51].

The statistical analyses were performed using the SAS software (SAS for Windows, version 9.4, SAS Institute Inc., Cary, NC). Statistical significance was set at a 2-sided p=0.05.

## SUPPLEMENTARY MATERIALS FIGURES AND TABLES







## Abbreviations

CAPOX: capecitabine and oxaliplatin
CI: confidence interval
CRC: colorectal cancer
DFS: disease-free survival
FFPE: Formalin-Fixed Paraffin-Embedded
FOLFOX: oxaliplatin, leucovorin and 5-fluoruracil
HeCOG: Hellenic Cooperative Oncology Group
IHC: immunohistochemistry
HPF: high power field
HR: hazard ratio
i-CD8: intratumoral CD8
LVI: lymphovascular invasion
MMR: mismatch repair
MMR-D: MMR deficient
MMR-P: MMR proficient
N: number
NGS: next generation sequencing
OS: overall survival
PNI: perineural invasion
s-CD8: stromal CD8
SD: standard deviation
TIL: tumor-infiltrating lymphocyte
TMA: tissue microarray
VAF: variant allele frequency
