# Supplementary material for: Prognostic significance of tumor genotypes and CD8+ infiltrates in stage I-III colorectal cancer
Source: Oncotarget. 2018 Nov 2;9(86):35623–38. doi: 10.18632/oncotarget.26256 (PMC6235022; doi:10.18632/oncotarget.26256)
Supplement: Supplementary file 1 [file oncotarget-09-35623-s001.pdf]

Prognostic significance of tumor genotypes and CD8+ infiltrates in stage I-III colorectal cancer

SUPPLEMENTARY MATERIALS

Immunohistochemistry (IHC)

We applied IHC on 3um TMA sections. All protocols were carried out with a Bond Max autostainer (Leica Microsystems, Wezlar, Germany) using Bond Polymer Refine Detection kit DS9800 (Bond Max and Bond III autostainers (Leica Microsystems, Wezlar, Germany) according to the manufacturer's instructions. The antibodies and staining protocols used are presented in the Table below:

Antibody*	Clone	Code	Staining protocol**
CD8	C8-144B	M7103	20min ER2, Ab 1:80, 20min
MLH1	ES05	M3640	20min ER2, Ab 1:50, 30min
MSH2	FE11	M3639	20min ER2, Ab 1:30, 30min
MSH6	EP49	M3646	20min ER2, Ab 1:70, 30min
PMS2	EP51	M3647	20min ER2, Ab 1:60, 30min

**: all antibodies from DAKO, Glostrup, DK*

***: Bond Polymers for all protocols*

ER1: citric acid; ER2: EDTA

NGS genotyping

DNA was extracted from 8 X 10um thick TMA core sections with the QIAamp DNA Mini Kit (Qiagen GmbH, Hilden, Germany). A total of 412 FFPE DNA samples were prepared. DNA quantity was measured with the Qubit fluorometer (Life Technologies, Paisley, UK). Samples were not processed for NGS if DNA quantity was <2 ng/ul upon vacuum concentration to a minimum volume of 11ul. Based on this criterion, we processed DNA samples from 404 tumors.

We performed targeted NGS with a custom Ampliseq panel (Applied Biosystems/Ion Torrent/ Thermo – Fisher Scientific, Paisley, UK), designed primarily for usage with FFPE DNA. The panel is described in Supplementary Table 1; targets included TCGA reported mutations in CRC, as well as coding regions of mismatch repair (MMR) and homologous recombination (HRR) genes. The design was based on GRCh37 (hg19) reference DNA and finally included 1271 amplicons, targeting a total region of approximately 120.8Kb from 55 protein encoding genes and 4 miRNAs. Ampliseq designed primers were evaluated for specificity (BLAST). For library construction, a multiplex PCR was performed using 20 ng DNA per sample and the Ampliseq primers along with the Ion AmpliSeq™ Library Kit 2.0 and Ion Xpress barcodes, according to the manufacturer's instructions (Life Technologies, Carlsbad, CA). Sample libraries, once normalized to 15 ng/ml with the Qubit fluorometer (Thermo, Fisher Scientific, Waltham, MA) were processed on a One-Touch-2 instrument followed by enrichment on a One-Touch-ES station. Templating was carried out using the Ion PI™ Hi-Q™ OT2 200 Kit, whereas sequencing of up to 48 sample libraries was performed with the Ion PI™ Hi-Q™ Sequencing 200 Kit on PI chips (Life Technologies, Carlsbad, CA) in an Ion Proton sequencer.

NGS data (base calling and the generation of sequence reads) were processed on the Torrent Server using Torrent Suite v.5, followed by adapter sequence trimming, read alignment to the human reference genome and variant calling. Annotated variants were retrieved from Ion Reporter v.5, raw annotated data were evaluated for the reads of all amplicons in the panel (provided by the embedded coverage analysis plug-in) and further quality filtered for accepting eligible variants. We applied the following steps for data quality control (QC):

(i). Panel evaluation. The number of minimum required reads per amplicon was set at 100. Out of 1271 amplicons, 23 (1.8%) did not meet this criterion in any sample and were excluded from further analysis.

(ii). Sample evaluation. Samples were excluded from further analysis if: mapped reads <130000; mean depth <150X; uniformity <50%; number of variants <30; number of sequenced amplicons with coverage <100 among all eligible amplicons (threshold for acceptance: 500 eligible amplicons/sample). The mean depth threshold was set at 450X for samples without detected mutations.

(iii). Variant evaluation. Variants were ineligible for analysis if: duplicates; single nucleotide variants with amplicon and position reads <100 and indels with amplicon reads <200; variant coverage <40 for single nucleotide changes and <80 for small indels; p-value > 0.0001 (metric including read quality and false discovery rate); GC stretches; strand bias (+/- <10% for both position and variant coverage).

Based on the above criteria, we excluded 57 tumors (86% NGS efficiency).

Variant characterization

Variants were distinguished into: (a) common single nucleotide polymorphisms (SNPs), with average minor allele frequency (MAF) >0.1% taken from GO-

ESP, ExAC and 1000 Genomes, along with a valid dbSNP string; (b) mutations, for genetic variations in coding and adjacent intronic regions resulting in amino acid or splice site change (non-synonymous), with MAF <0.1%, based on the aforementioned sources. We further categorized mutations as clonal (>25% variant allele frequency [VAF] (McGranahan, Favero et al. 2015)) and as pathogenic, non-pathogenic (benign and of unknown significance) and of unknown function (no information could be found). Pathogenic mutations were returned as such by Ion Reporter with FATHMM, ClinVar and COSMIC (confirmed in each case); or, these were non-annotated, nonsense and frameshift mutations in tumor suppressor genes with >100 variant coverage, with clonal VAFs for the sample, i.e., >25% VAF in samples with >50% TCC, or >15% VAF in samples with <50% TCC. Position loss (LOH) was inferred for VAFs (mutated allele) >65%. Non-annotated variants were not evaluated for pathogenic status if they were G>A/C>T of non-clonal VAF according to TCC% as aforementioned, or if they were inadequately covered (<400 for the position or <100 for the variant).

Informative tumors were read at very high depth (technical characteristics and variant distribution are shown in Supplementary Figure 3). Out of ~50000 variants, we processed ~35000 for analysis, their number varying from 30 – 690 variants per tumor (mean 101; median 76). We identified 5231 amino acid or splice site changing mutations in 55 out of the 59 genes of our panel. Mutations (range 1 – 220; median 4; mean±SD 15±35) were distributed in 339/347 informative tumors (97.7%). Compared to non-pathogenic, pathogenic mutations exhibited significantly higher VAFs and differed in the distribution of functional types (both p's <0.0001; Supplementary Figure 4) including 64% missense, 24% nonsense and 8% frameshifts. Non-pathogenic mutations included 94% missense almost exclusively G>A/C>T changes that would be impossible to validate individually at VAFs <12%. For this reason, we next decided to analyze pathogenic mutations only, although pathogenicity inferred from the germline does not necessarily apply to the behaviour of the same changes in tissues.

REFERENCES

1. McGranahan N, Favero F, de Bruin EC, Birkbak NJ, Szallasi Z, Swanton C. Clonal status of actionable driver events and the timing of mutational processes in cancer evolution. Sci Transl Med. 2015; 7:283ra54.

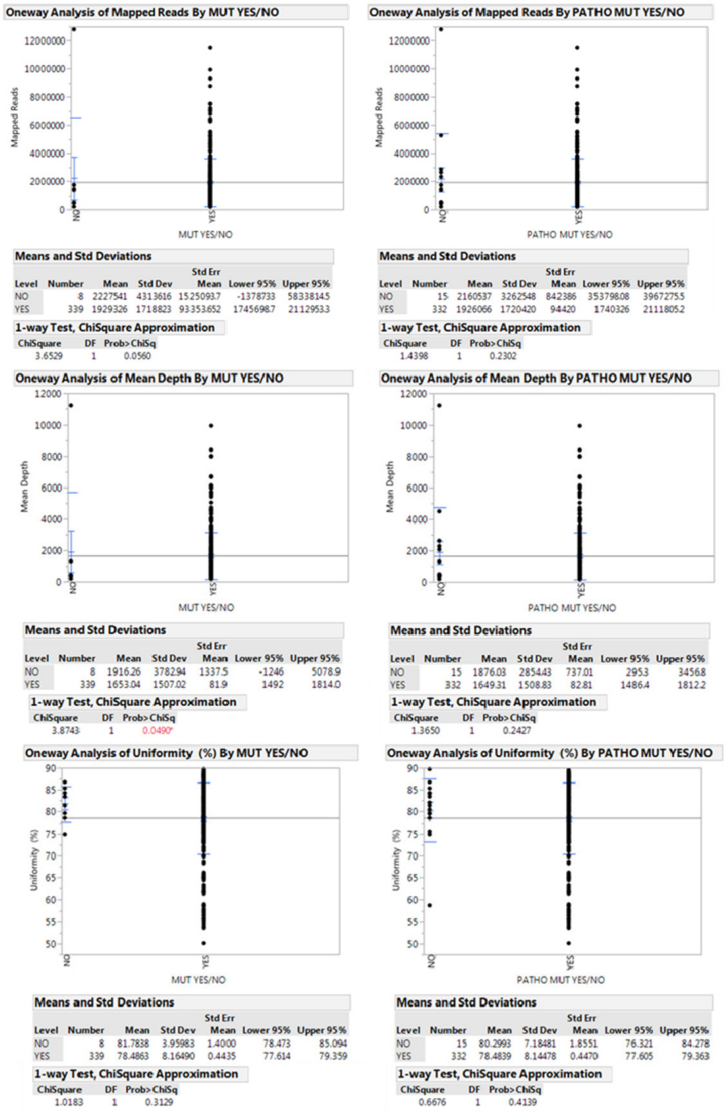

Supplementary Figure 1: Technical characteristics of the 8 tumors without any detected mutation with the Ampliseq panel and of the 15 tumors without pathogenic mutations. As shown in the graphs above, in comparison to tumors with mutations, tumors without mutations (left panel) and those without pathogenic mutations (right panel) had similar or slightly better technical performance metrics.

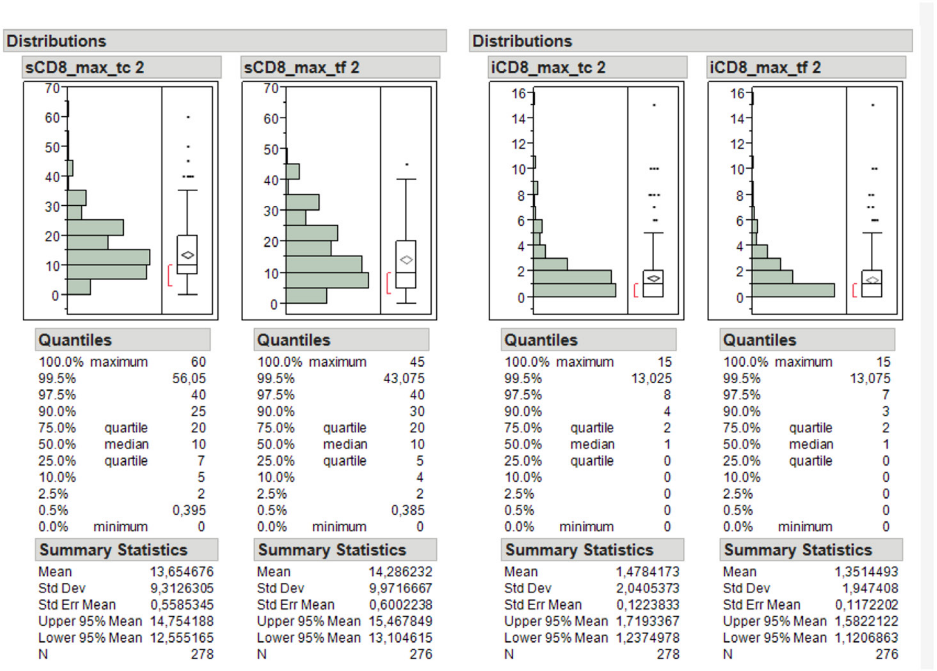

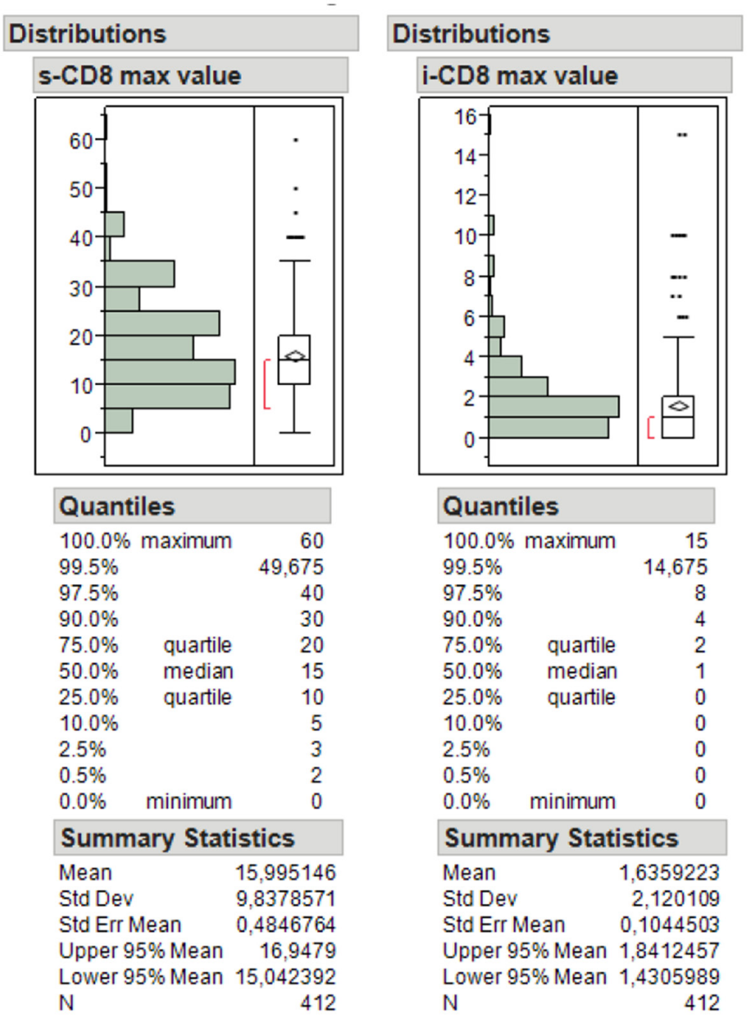

Supplementary Figure 2: CD8 IHC evaluation. **(A)** Similar distributions of maximal s-CD8 and i-CD8 counts were observed in the tumor center (tc) and the tumor invasive front (tf) in the 276 and 278 cases with available corresponding data. **(B)** Distribution of merged maximal s-CD8 and i-CD8 counts in all examined tumors.

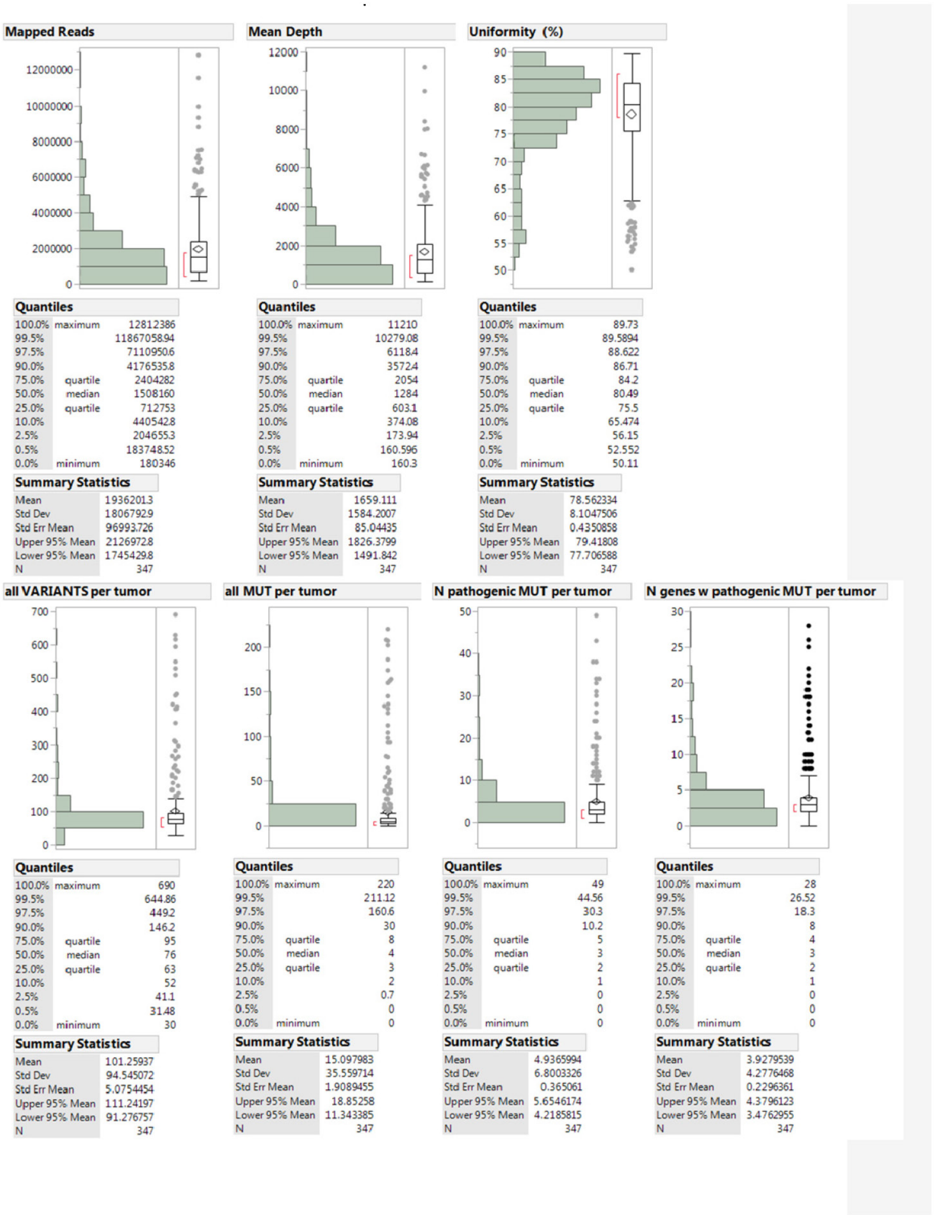

Supplementary Figure 3: Technical characteristics and variant distribution in the 347 NGS informative FFPE tumor samples.

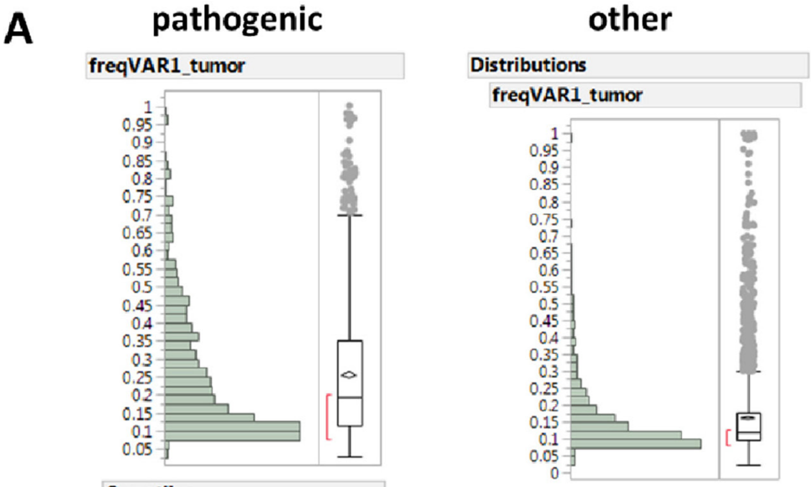

pathogenic — Quantiles

100.0%	maximum	1
99.5%		0.94131084883
97.5%		0.739220091075
90.0%		0.4975693815
75.0%	quartile	0.35209339775
50.0%	median	0.195702162
25.0%	quartile	0.11621558925
10.0%		0.0926514823
2.5%		0.0797220628
0.5%		0.07169835527
0.0%	minimum	0.031148605

Summary Statistics

Mean	0.2552855
Std Dev	0.1776116
Std Err Mean	0.0044128
Upper 95% Mean	0.2639409
Lower 95% Mean	0.2466301

other — Quantiles

100.0%	maximum	1
99.5%		0.79592469902
97.5%		0.5057513071
90.0%		0.2838492558
75.0%	quartile	0.17768619175
50.0%	median	0.1207275735
25.0%	quartile	0.096612296
10.0%		0.085106383
2.5%		0.076242494925
0.5%		0.04418695767
0.0%	minimum	0.024549098

Summary Statistics

Mean	0.1618525
Std Dev	0.1167547
Std Err Mean	0.0019432
Upper 95% Mean	0.1656624
Lower 95% Mean	0.1580426

B

Count<br>Row %	Other	Pathogenic	
Frameshift	21<br>13.2	138<br>86.8	159
Missense	3312<br>75.3	1102<br>24,9	4414
Nonframeshift	1<br>50.0	1<br>50.0	2
Nonsense	110<br>20.5	425<br>79.4	535
Unknown	82<br>63.6	47<br>36.4	129
	3526	1713	5239

Tests

N	DF	-LogLike	RSquare (U)
5240	4	411,62785	0,1243

Test	ChiSquare	Prob>ChiSq
Likelihood Ratio	823,256	<,0001*
Pearson	865,266	<,0001*

Supplementary Figure 4: Comparison of all mutations and pathogenic mutations. Pathogenic mutations were present at higher VAFs **(A)** and were more frequently frameshifts and nonsense **(B)** compared to non-pathogenic mutations (all p's <0.0001).

Supplementary Table 1: Clinicopathological, immunophenotypic and genotypic parameters in association with MMR status

Parameters*	MMR		
	Deficiency	Proficiency	p-value
**Age**			**0.010**
Mean (SD)	59 (12)	64 (10)	
Median (IQR)	61 (49-69)	65 (57-72)	
Min-Max	38-76	28-80	
**Gender**			0.681
Female	17 (42.5)	147 (45.9)	
Male	23 (57.5)	173 (54.1)	
**Family history of cancer**			0.99
No	12 (54.5)	111 (54.7)	
Yes	10 (45.5)	92 (45.3)	
**Stage (excl. I)**			**0.041**
II	19 (52.8)	109 (35.4)	
III	17 (47.2)	199 (64.6)	
**Grade**			**<0.001**
G1-G2	22 (57.9)	260 (82.8)	
G3	16 (42.1)	54 (17.2)	
**Mucinous component**			**0.008**
No	20 (55.6)	221 (76.2)	
Yes	16 (44.4)	69 (23.8)	
**Perineural invasion**			0.066
No	36 (94.7)	250 (83.3)	
Yes	2 (5.3)	50 (16.7)	
**Lympatic vessel invasion**			0.213
No	26 (68.4)	231 (77.5)	
Yes	12 (31.6)	67 (22.5)	
**Blood vessel invasion**			0.291
No	35 (92.1)	256 (85.9)	
Yes	3 (7.9)	42 (14.1)	
**PNI+/-LVI**			0.504
No	23 (60.5)	198 (66.0)	
Yes	15 (39.5)	102 (34.0)	
**CD8 density**			**<0.001**
High	26 (65.0)	85 (26.6)	
Other	14 (35.0)	235 (73.4)	
**Mutated tumors**			N/A
No	0 (0)	8 (3.0)	
Yes	35 (100.0)	257 (97.0)	
**Mutations per tumor**			**0.003**
Mean +/- SD	21.1 +/- 38.9	15.2 +/- 37.6	
Median (IQR)	6.0 (4.0, 18.0)	4.0 (2.0, 7.0)	
Min-Max	1-186	0-220	

(*Continued*)

Parameters*	MMR		
	Deficiency	Proficiency	p-value
**Pathogenic mutations**			0.092
Mean +/- SD	6.3 +/- 7.4	4.9 +/- 7.2	
Median (IQR)	3.0 (2.0, 6.0)	3.0 (2.0, 4.0)	
Min-Max	0-34	0-49	
**Mutated genes per tumor**			0.073
Mean +/- SD	4.9 +/- 4.6	3.9 +/- 4.5	
Median (IQR)	3.0 (2.0, 6.0)	3.0 (2.0, 4.0)	
Min-Max	0-18	0-28	
**MMR MUT**			0.323
No	31 (88.6)	248 (93.6)	
Yes	4 (11.4)	17 (6.4)^	
**BRAF**			**<0.001**
No	26 (74.3)	252 (95.1)	
Yes	9 (25.7)	13 (4.9)	
**ALL RAS**			0.441
No	18 (51.4)	118 (44.5)	
Yes	17 (48.6)	147 (55.5)	
**BRCA1**			0.682
No	31 (88.6)	228 (86.0)	
Yes	4 (11.4)	37 (14.0)	
**ARID1A**			0.187
No	29 (82.9)	239 (90.2)	
Yes	6 (17.1)	26 (9.8)	

IQR = Interquartile range

* analysis performed in informative samples

^ We did not include 5 MSH3-mutated tumors in the analysis, because the MSH3 protein was not tested with immunohistochemistry.

Supplementary Table 2: Clinicopathological characteristics and outcome in patients with BRAF p.V600E and non-V600E mutations

case	BRAF protein change	TCC%	mutation VAF	Stage	MMR status	CD8	LEFT_ RIGHT_ RECTUM	N mutations per tumor	OS	OS_ index	DFS	DFS_ index
1	p.Gln609Arg	48.8	0.40	III	Proficiency	low	RIGHT	5	84.03	0	84.03	0
2	p.Asn581Ser	46.3	0.47	III	Proficiency	high	RECTAL	2	73.28	0	73.28	0
3	p.Asp594Gly	65.0	0.20	III	Proficiency	high	LEFT	30	112.66	0	112.66	0
4	p.Arg444Gln	55.0	0.08	III	Proficiency	low	RECTAL	134	113.64	0	113.64	0
5	p.Val600Glu; p.Ala598Thr	50.0	0,16; 0,10	II	Deficiency	low	RECTAL	78	83.28	0	83.28	1
6	p.Val600Glu	60.0	0.11	III	not available	high	LEFT	4	110.89	0	110.89	0
7	p.Val600Glu	57.5	0.27	III	not available	high	LEFT	5	58.52	0	58.52	0
8	p.Val600Glu	76.7	0.36	III	not available	high	RIGHT	3	118.43	0	118.43	0
9	p.Val600Glu	72.5	0.20	III	Deficiency	high	RIGHT	3	76.66	0	76.66	0
10	p.Val600Glu	28.3	0.23	II	Deficiency	low	RIGHT	6	98.23	0	98.23	0
11	p.Val600Glu	66.3	0.24	III	Deficiency	high	RIGHT	6	70.33	0	70.33	0
12	p.Val600Glu	70.0	0.24	III	Deficiency	high	RIGHT	3	69.87	0	69.87	0
13	p.Val600Glu	62.5	0.31	II	Deficiency	high	RIGHT	3	97.18	0	97.18	0
14	p.Val600Glu	52.5	0.45	II	Deficiency	high	RIGHT	5	73.93	0	73.93	0
15	p.Val600Glu	25.0	0.18	II	Proficiency	low	RIGHT	3	103.11	0	103.11	0
16	p.Val600Glu	65.0	0.18	II	Proficiency	low	LEFT	6	37.31	0	37.31	0
17	p.Val600Glu	46.3	0.35	II	Proficiency	low	LEFT	136	106.52	0	106.52	0
18	p.Val600Glu	57.5	0.19	III	Proficiency	low	RIGHT	4	49.28	0	19.84	1
19	p.Val600Glu	52.5	0.27	III	Proficiency	low	RIGHT	4	60.36	0	25.44	1
20	p.Val600Glu	25.0	0.24	III	not available	low	LEFT	6	39.64	1	24.43	1
21	p.Val600Glu	38.8	0.16	III	Deficiency	low	RIGHT	5	71.77	1	71.77	1
22	p.Val600Glu	68.8	0.35	III	Deficiency	low	RIGHT	7	110.1	1	110.1	1
23	p.Val600Glu	42.5	0.18	III	Proficiency	high	RIGHT	2	17.64	1	8.59	1
24	p.Val600Glu	46.7	0.20	II	Proficiency	low	LEFT	4	60.85	1	60.85	1
25	p.Val600Glu	10.0	0.23	III	Proficiency	low	RECTAL	6	13.7	1	7.02	1
26	p.Val600Glu	80.0	0.40	III	Proficiency	high	RIGHT	3	21.21	1	14.72	1

TCC: tumor cell content; VAF: variant allelic frequency.

Supplementary Table 3: Clinicopathological characteristics of tumors with MMR gene mutations compared to those classified as MMR-D with IHC

case	location	MMR status	TCC%	MLH1	protein variant	VAF	PMS2	protein variant	VAF	MSH2	protein variant	VAF	MSH6	protein variant	VAF	N mutations	other clonal pathogenic
1	right	deficient; MLH1; MSH2	56.3		splice site c.790+1G>A	0.44					p.Gln183Ter; p.Pro439Leu	0,10; 0,14				187	BRCA1, splicing; c.4185+1G>A
2	rectal	deficient; MLH1	35.0		p.Asp41Val; p.Phe88fs	0,63; 0,20										18	none
3	right	deficient; MSH2	70.0								p.Gln413Ter	0.46				9	KRAS; PIK3CA; TP53; PTEN; RB1
4	left	deficient; MSH6	46.3											p.Gln1146Ter	0.32	30	POLE p.Ser297Phe
5	left	proficient	50.0		p.Arg127Lys	0.12					p.Gln681Ter	0.31				220	BRCA1, splicing; c.81-1G>A; APC c.3927_3931delAAAGA
6	left	proficient	45.0		p.His109Tyr	0.08										173	KRAS p.Gly12Val; TP53 p.Pro190Leu
7	rectal	proficient	42.5		p.Thr117Met	0.11										207	ARID1A; TP53; FBXW7; RNF43; KMT2C; PALB2
8	left	proficient	35.0					p.Gln400Ter	0.21		p.Pro696Leu	0.12				162	POLE p.Pro286Leu; PALB2 p.Gln1056Ter
9	right	proficient	30.0								splice site c.1760-1G>A	0.10				208	BRCA1 p.Ser988Ter; POLE p.Asp301Asn
10	right	proficient	55.0								p.Arg621Gln	0.13				9	MSH3 p.Ala177Thr
11	left	proficient	72.5								p.Gln170Ter	0.09				30	none
12	left	proficient	46.3								p.Gln193Ter	0.47				202	BRCA1 p.Gly1759Arg; ARID1A p.Gly410Arg; MSH3 p.Trp1111Ter; PALB2 p.Arg753Ter
13	left	proficient	38.8								p.Glu749Ter	0.12				13	POLE p.Pro286Arg; TP53 p.Arg213Ter
14	left	proficient	51.7								p.Gly164Arg	0.12				43	APC p.Lys1308Ter; TP53 p.Tyr234Cys
15	left	proficient	27.5											p.Trp912Ter	0.34	160	POLE p.Ala480Val; ARID1A p.Gly960Glu; NRAS; TP53; SMAD4
16	rectal	proficient	63.3											p.Gln132Ter	0.29	93	CDKN2A c.457+1G>A; BRCA2 p.Glu1593Lys; PTEN; NRAS; TP53
17	rectal	proficient	55.0											p.Glu286Lys	0.13	134	POLE p.Pro436Ser; TP53 p.Pro27fs; APC p.Glu1309fs
18	right	deficient	45.0													5	none
19	right	deficient	38.3													8	KRAS p.Ala146Val
20	right	deficient	70.0													9	ARID1A; CDH1; FBXW7; KRAS; SMAD4
21	left	deficient	43.3													4	BRCA2 p.Asp2723Gly
22	right	deficient	55.0													25	APC; KRAS; PIK3CA; TP53
23	right	deficient	53.3													3	APC; KRAS; PIK3CA
24	right	deficient	36.7													4	TP53 p.Arg342Ter
25	right	deficient	83.3													4	CDH1 p.Arg335Ter
26	right	deficient	72.5													3	CDKN1B p.Lys73fs; RNF43 p.Gly659fs
27	rectal	deficient	50.0													78	BRCA1 p.Gly1759Arg; BRCA2 p.Arg2659Lys
28	right	deficient	52.5													5	BRAF p.Val600Glu; NBN p.Arg89Ter; RNF43 p.Gly659fs
29	right	deficient	66.3													6	BRAF p.Val600Glu; RNF43 p.Gly659fs
30	right	deficient	65.0													4	APC p.Glu1156fs
31	right	deficient	70.0													3	BRAF p.Val600Glu; PTEN p.Gly251Val
32	left	deficient	71.3													9	CHEK2 p.Arg95Ter; CTNNB1 p.Ser45Phe
33	right	deficient	52.5													93	BRCA1 p.Trp1529Ter; PALB2 p.Gln251Ter; + 9 additional genes
34	right	deficient	38.8													5	none
35	right	deficient	66.7													4	ERBB4 p.Pro224His

(*Continued*)

case	location	MMR status	TCC%	MLH1	protein variant	VAF	PMS2	protein variant	VAF	MSH2	protein variant	VAF	MSH6	protein variant	VAF	N mutations	other clonal pathogenic
36	right	deficient	57.5													3	APC p.Arg876Ter; KMT2D p.Arg5501Ter; TP53 p.Arg273Cys
37	right	deficient	77.5													1	BRCA2 p.Asp2723Gly
38	right	deficient	28.3													6	RNF43 p.Gly659fs
39	right	deficient	62.5													3	BRAF p.Val600Glu; PTEN p.Lys267fs
40	right	deficient	52.5													44	BRCA1 p.Gln1832Ter
41	rectal	deficient	60.0													3	APC p.Ile1557Ter; KRAS p.Gln61His; TP53 p.Lys132Asn
42	right	deficient	23.8													112	ARID1A p.Gln403Ter; MYC p.Glu384Lys
43	left	deficient	48.8													5	NBN p.Asn209fs; RNF43 p.Gly659fs; FBXW7 p.Arg479Gln
44	right	deficient	68.8													7	BRAF p.Val600Glu + 2 different PTEN stogains (nonsense)
45	right	deficient	40.0													6	TP53 p.Arg213Ter + APC, KRAS, PIK3CA
46	right	deficient	53.3													24	KRAS p.Gly12Asp
47	right	deficient	43.8													5	KRAS p.Gln61His; PIK3CA p.His1047Arg; TP53 p.Leu43Ter
48	right	deficient	43.3													6	KRAS p.Gly12Asp; SMAD4 p.Arg361Ser; TP53 p.Ser127Tyr

TCC: tumor cell content; VAF: variant allelic frequency.

Supplementary Table 4: Clinicopathological, immunophenotypic and genotypic parameters in association with ARID1A and BRCA1 mutations

Parameters*	ARID1A			BRCA1		
	Wt	Mut	p-value	Wt	Mut	p-value
**Age**						
Mean (SD)	63 (10)	61 (12)	0.390	63 (11)	62 (11)	0.330
Median (IQR)	65 (57-72)	62 (52-70)		65 (56-72)	57-69	
Min-Max	28-81	30-79		28-81	28-78	
**Gender**						
Female	145 (46.6)	11 (30.6)	0.067	138 (45.8)	18 (39.1)	0.394
Male	166 (53.4)	25 (69.4)		163 (54.2)	28 (60.9)	
**Family history of cancer**						
No	114 (58.8)	11 (42.3)	0.112	108 (58.1)	17 (50.0)	0.383
Yes	80 (41.2)	15 (57.7)		78 (41.9)	17 (50.0)	
**Stage (excl. I)**						
II	94 (31.6)	13 (37.1)	0.511	87 (30.3)	20 (44.4)	0.059
III	203 (68.4)	22 (62.9)		200 (69.7)	25 (55.6)	
**Grade**						
G1-G2	238 (79.1)	28 (80.0)	0.898	231 (79.1)	35 (79.5)	0.947
G3	63 (20.9)	7 (20.0)		61 (20.9)	9 (20.5)	
**Mucinous component**						
No	213 (74.7)	17 (51.5)	**0.005**	205 (73.7)	25 (62.5)	0.137
Yes	72 (25.3)	16 (48.5)		73 (26.3)	15 (37.5)	
**Perineural invasion**						
No	248 (84.9)	32 (88.9)	0.526	248 (86.4)	32 (78.0)	0.156
Yes	44 (15.1)	4 (11.1)		39 (13.6)	9 (22.0)	
**Lympatic vessel invasion**						
No	226 (77.4)	29 (80.6)	0.667	225 (78.4)	30 (73.2)	0.452
Yes	66 (22.6)	7 (19.4)		62 (21.6)	11 (26.8)	
**Blood vessel invasion**						
No	252 (86.6)	31 (86.1)	0.936	249 (87.1)	34 (82.9)	0.468
Yes	39 (13.4)	5 (13.9)		37 (12.9)	7 (17.1)	
**PNI+/-LVI**						
No	191 (65.0)	26 (72.2)	0.386	192 (66.4)	25 (61.0)	0.49
Yes	103 (35.0)	10 (27.8)		97 (33.6)	16 (39.0)	
**CD8 density**						
High	83 (26.7)	13 (36.1)	0.232	83 (27.6)	13 (28.3)	0.923
Other	228 (73.3)	23 (63.9)		218 (72.4)	33 (71.7)	
**MMR**						
Deficiency	29 (10.8)	6 (18.8)	0.187	31 (12.0)	4 (9.8)	0.682
Proficiency	239 (89.2)	26 (81.3)		228 (88.0)	37 (90.2)	
**Mutated tumors**						
No	8 (2.6)	0 (0)	N/A	8 (2.7)	0 (0)	N/A
Yes	303 (97.4)	36 (100.0)		293 (97.3)	46 (100.0)	

(*Continued*)

Parameters*	ARIDIA			BRCA1		
	Wt	Mut	p-value	Wt	Mut	p-value
**Mutations per tumor**						
Mean +/- SD	8.3 +/- 20.1	73.8 +/- 70.3	**<0.001**	7.6 +/- 16.5	64.2 +/- 71.2	**<0.001**
Median (IQR)	4.0 (3.0, 6.0)	43.5 (8.5, 128.5)		4.0 (3.0, 6.0)	36.5 (6.0, 126.0)	
Min-Max	0-208	3-220		0-160	2-220	
**Pathogenic mutations**						
Mean +/- SD	3.7 +/- 4.3	16.0 +/- 12.2	**<0.001**	3.4 +/- 3.4	14.7 +/- 12.9	**<0.001**
Median (IQR)	3.0 (2.0, 4.0)	12.0 (5.0, 25.0)		3.0 (2.0, 4.0)	11.0 (4.0, 24.0)	
Min-Max	0-49	2-43		0-34	1-49	
**Mutated genes per tumor**						
Mean +/- SD	3.1 +/- 2.8	11.1 +/- 7.1	**<0.001**	3.0 +/- 2.4	10.2 +/- 7.5	**<0.001**
Median (IQR)	3.0 (2.0, 3.0)	9.5 (4.5, 16.5)		3.0 (2.0, 3.0)	8.0 (3.0, 17.0)	
Min-Max	0-25	2-28		0-19	1-28	
**MMR MUT**						
No	293 (94.2)	27 (75.0)	**<0.001**	286 (95.0)	34 (73.9)	**<0.001**
Yes	18 (5.8)	9 (25.0)		15 (5.0)	12 (26.1)	
**BRAF**						
No	289 (92.9)	32 (88.9)	0.384	278 (92.4)	43 (93.5)	0.788
Yes	22 (7.1)	4 (11.1)		23 (7.6)	3 (6.5)	
**ALL RAS**						
No	141 (45.3)	14 (38.9)	0.461	135 (44.9)	20 (43.5)	0.862
Yes	170 (54.7)	22 (61.1)		166 (55.1)	26 (56.5)	

IQR = Interquartile range, SD standard deviation.

* analysis performed in informative samples.

Supplementary Table 5: Prognostic significance of clinicopathological parameters, in the entire cohort and per tumor location (DFS)

Parameter	Categories	Npatients	Nevents	HR	95% CI	Wald's p
			**Entire cohort**			
**Age (median cut-off)**	<Median vs. ≥Median	203 vs. 209	51 vs. 62	0.82	0.56-1.18	0.28
**Gender**	Female vs. Male	183 vs. 229	45 vs. 68	0.79	0.54-1.15	0.22
**Tumor location (Left-Right-Rectal)**						0.63
	Left vs. right	171 vs. 139	50 vs. 39	1.03	0.68-1.56	0.9
	Rectal vs. right	102 vs. 139	24 vs. 39	0.82	0.49-1.36	0.43
**Perforation**	No vs. Yes	375 vs. 14	102 vs. 5	0.68	0.28-1.68	0.4
**Obstruction**	No vs. Yes	345 vs. 45	91 vs. 16	0.71	0.42-1.21	0.21
**Radiation therapy**	No vs. Yes	279 vs. 84	75 vs. 25	0.91	0.58-1.43	0.68
**Primary tumor (T)**						**0.014**
	T1-T2 vs. T4	39 vs. 41	5 vs. 17	0.24	0.09-0.65	0.005
	T3 vs. T4	329 vs. 41	90 vs. 17	0.59	0.35-0.99	0.046
**Regional lymph nodes (N)**	N0 vs. N1-2	151 vs. 249	25 vs. 84	0.43	0.28-0.67	**<0.001**
**Stage (excluding I)**	II vs. III	137 vs. 258	24 vs. 87	0.46	0.29-0.72	**0.001**
**Treatment**	FOLFOX vs. CAPOX	137 vs. 270	40 vs. 71	1.1	0.75-1.62	0.63
**Grade**	G1-G2 vs. G3	320 vs. 79	81 vs. 28	0.68	0.45-1.05	0.083
**Mucinous component**	No vs. Yes	273 vs. 102	70 vs. 31	0.8	0.52-1.22	0.3
**Perineural invasion (PNI)**	No vs. Yes	328 vs. 59	86 vs. 19	0.77	0.47-1.27	0.3
**Lympatic vessel invasion**	No vs. Yes	298 vs. 89	78 vs. 28	0.84	0.54-1.29	0.41
**Blood vessel invasion**	No vs. Yes	332 vs. 54	82 vs. 23	0.51	0.32-0.82	**0.005**
**PNI+/-LVI**	No vs. Yes	253 vs. 136	61 vs. 46	0.69	0.47-1.02	**0.062**
			**Right (N=139)**			
**Age (median cut-off)**	<Median vs. ≥Median	70 vs. 69	20 vs. 19	1.09	0.58-2.04	0.79
**Gender**	Female vs. Male	62 vs. 77	16 vs. 23	0.8	0.42-1.51	0.49
**Perforation**	No vs. Yes	129 vs. 2	38 vs. 0	-	-	-
**Obstruction**	No vs. Yes	118 vs. 13	33 vs. 5	0.64	0.25-1.63	0.35
**Radiation therapy**	No vs. Yes	119 vs. 1	34 vs. 1	0.16	0.02-1.18	0.072
**Primary tumor (T)**						0.48
	T1-T2 vs. T4	8 vs. 14	1 vs. 5	0.28	0.03-2.42	0.25
	T3 vs. T4	117 vs. 14	33 vs. 5	0.68	0.27-1.76	0.43
**Regional lymph nodes (N)**	N0 vs. N1-2	55 vs. 81	9 vs. 30	0.36	0.17-0.76	0.007
**Stage (excluding I)**	II vs. III	49 vs. 84	7 vs. 31	0.31	0.14-0.70	0.005
**Treatment**	FOLFOX vs. XELOX	40 vs. 98	12 vs. 27	1.05	0.53-2.08	0.88
**Grade**	G1-G2 vs. G3	93 vs. 41	24 vs. 15	0.71	0.37-1.36	0.3
**Mucinous component**	No vs. Yes	84 vs. 41	24 vs. 12	0.93	0.46-1.86	0.84
**Perineural invasion**	No vs. Yes	110 vs. 16	30 vs. 6	0.68	0.28-1.65	0.4
**Lympatic vessel invasion**	No vs. Yes	92 vs. 34	26 vs. 11	0.86	0.42-1.74	0.67
**Blood vessel invasion**	No vs. Yes	109 vs. 17	29 vs. 8	0.49	0.22-1.07	0.072
**PNI+/-LVI**	No vs. Yes	78 vs. 49	19 vs. 18	0.62	0.32-1.18	0.14
			**Left (N=171)**			
**Age (median cut-off)**	<Median vs. ≥Median	77 vs. 94	19 vs. 31	0.7	0.40-1.24	0.22
**Gender**	Female vs. Male	77 vs. 94	15 vs. 35	0.48	0.26-0.87	0.017
**Perforation**	No vs. Yes	151 vs. 11	42 vs. 4	0.63	0.23-1.77	0.39
**Obstruction**	No vs. Yes	135 vs. 27	36 vs. 10	0.73	0.36-1.47	0.37
**Radiation therapy**	No vs. Yes	147 vs. 4	40 vs. 2	0.47	0.11-1.96	0.3
**Primary tumor (T)**						0.17
	T1-T2 vs. T4	14 vs. 20	2 vs. 9	0.27	0.06-1.25	0.094
	T3 vs. T4	136 vs. 20	38 vs. 9	0.58	0.28-1.20	0.14

(*Continued*)

Parameter	Categories	Npatients	Nevents	HR	95% CI	Wald's p
**Regional lymph nodes (N)**	N0 vs. N1-2	59 vs. 105	10 vs. 36	0.45	0.23-0.92	0.027
**Stage (excluding I)**	II vs. III	54 vs. 110	11 vs. 38	0.56	0.29-1.10	0.09
**Treatment**	FOLFOX vs. CAPOX	64 vs. 104	18 vs. 30	1.01	0.56-1.80	0.98
**Grade**	G1-G2 vs. G3	137 vs. 29	38 vs. 9	0.86	0.42-1.79	0.69
**Mucinous component**	No vs. Yes	120 vs. 37	31 vs. 13	0.7	0.37-1.34	0.28
**Perineural invasion**	No vs. Yes	135 vs. 28	35 vs. 11	0.61	0.31-1.19	0.15
**Lympatic vessel invasion**	No vs. Yes	129 vs. 34	35 vs. 11	0.86	0.43-1.69	0.65
**Blood vessel invasion**	No vs. Yes	136 vs. 26	33 vs. 12	0.47	0.24-0.90	0.024
**PNI+/-LVI**	No vs. Yes	107 vs. 57	26 vs. 21	0.64	0.36-1.15	0.13
			**Rectal (N=102)**			
**Age (median cut-off)**	<Median vs. ≥Median	56 vs. 46	12 vs. 12	0.73	0.33-1.64	0.45
**Gender**	Female vs. Male	44 vs. 58	14 vs. 10	2.05	0.91-4.61	0.084
**Perforation**	No vs. Yes	95 vs. 1	22 vs. 1	0.12	0.01-0.90	0.04
**Obstruction**	No vs. Yes	92 vs. 5	22 vs. 1	1.12	0.15-8.31	0.91
**Radiation therapy**	No vs. Yes	13 vs. 79	1 vs. 22	0.32	0.04-2.36	0.26
**Primary tumor (T)**						0.17
	T1-T2 vs. T4	17 vs. 7	2 vs. 3	0.18	0.03-1.07	0.06
	T3 vs. T4	76 vs. 7	19 vs. 3	0.45	0.13-1.53	0.2
**Regional lymph nodes (N)**	N0 vs. N1-2	37 vs. 63	6 vs. 18	0.5	0.20-1.27	0.15
**Stage (excluding I)**	II vs. III	34 vs. 64	6 vs. 18	0.55	0.22-1.40	0.21
**Treatment**	FOLFOX vs. XELOX	33 vs. 68	10 vs. 14	1.41	0.63-3.18	0.4
**Grade**	G1-G2 vs. G3	90 vs. 9	19 vs. 4	0.42	0.14-1.23	0.11
**Mucinous component**	No vs. Yes	69 vs. 24	15 vs. 6	0.83	0.32-2.14	0.7
**Perineural invasion**	No vs. Yes	83 vs. 15	21 vs. 2	1.99	0.47-8.48	0.35
**Lympatic vessel invasion**	No vs. Yes	77 vs. 21	17 vs. 6	0.82	0.32-2.09	0.68
**Blood vessel invasion**	No vs. Yes	87 vs. 11	20 vs. 3	0.74	0.22-2.51	0.63
**PNI+/-LVI**	No vs. Yes	68 vs. 30	16 vs. 7	1.05	0.43-2.54	0.92

Supplementary Table 6: Multivariate analysis of clinical parameters (DFS)

Parameter	Categories	N patients	N events	HR	95% CI	p-value
Stage	II vs. III	129 vs. 234	22 vs. 79	0.46	0.28-0.74	**0.001**
Grade	G1-G2 vs. G3	294 vs. 69	74 vs. 27	0.71	0.45-1.12	0.14
Blood vessel invasion	No vs. Yes	311 vs. 52	79 vs. 22	0.6	0.37-0.97	**0.037**
Tumor site	Left vs. right	154 vs. 117	43 vs. 36	0.85	0.54-1.33	0.46
	Rectal vs. right	92 vs. 117	22 vs. 36	0.8	0.46-1.38	0.8

Supplementary Table 7: Prognostic significance of molecular parameters, in the entire cohort and per tumor location (DFS)

See Supplementary File 1

Supplementary Table 8: 59-gene panel

See Supplementary File 2
